# Remote sampling of biomarkers of inflammation with linked patient generated health data in patients with rheumatic and musculoskeletal diseases: an Ecological Momentary Assessment feasibility study

**DOI:** 10.1186/s12891-022-05723-w

**Published:** 2022-08-13

**Authors:** Katie L. Druce, David S. Gibson, Kevin McEleney, Belay B. Yimer, Stephanie Meleck, Ben James, Bruce Hellman, William G. Dixon, John McBeth

**Affiliations:** 1 Centre for Epidemiology Versus Arthritis, Division of Musculoskeletal and Dermatological Sciences, The University of Manchester, Manchester, UK; 2 Manchester Academic Health Science Centre (MAHSC), University of Manchester, Manchester, UK; 3 Northern Ireland Centre for Stratified Medicine, School of Biomedical Sciences, Biomedical Sciences Research Institute, Ulster University, Londonderry, UK; 4 uMotif, London, UK; 5 The NIHR Manchester Musculoskeletal Biomedical Research Unit, Central Manchester University Hospitals NHS Foundation Trust, Manchester, UK

**Keywords:** Rheumatoid arthritis, Fibromyalgia, Osteoarthritis, Fatigue, Inflammation, mHealth, Ecological momentary assessment

## Abstract

**Background:**

People with rheumatic diseases experience troublesome fluctuations in fatigue. Debated causes include pain, mood and inflammation. To determine the relationships between these potential causes, serial assessments are required but are methodologically challenging. This mobile health (mHealth) study explored the viability of using a smartphone app to collect patient-reported symptoms with contemporaneous Dried Blood Spot Sampling (DBSS) for inflammation.

**Methods:**

Over 30 days, thirty-eight participants (12 RA, 13 OA, and 13 FM) used uMotif, a smartphone app, to report fatigue, pain and mood, on 5-point ordinal scales, twice daily. Daily DBSS, from which C-reactive Protein (CRP) values were extracted, were completed on days 1–7, 14 and 30. Participant engagement was determined based on frequency of data entry and ability to calculate within- and between-day symptom changes. DBSS feasibility and engagement was determined based on the proportion of samples returned and usable for extraction, and the number of days between which between-day changes in CRP which could be calculated (days 1–7).

**Results:**

Fatigue was reported at least once on 1085/1140 days (95.2%). Approximately 65% of within- and between-day fatigue changes could be calculated. Rates were similar for pain and mood. A total of 287/342 (83.9%) DBSS, were returned, and all samples were viable for CRP extraction. Fatigue, pain and mood varied considerably, but clinically meaningful (≥ 5 mg/L) CRP changes were uncommon.

**Conclusions:**

Embedding DBSS in mHealth studies will enable researchers to obtain serial symptom assessments with matched biological samples. This provides exciting opportunities to address hitherto unanswerable questions, such as elucidating the mechanisms of fatigue fluctuations.

**Supplementary Information:**

The online version contains supplementary material available at 10.1186/s12891-022-05723-w.

## Background

Approximately 75% of people with rheumatic diseases (RMD), including rheumatoid arthritis (RA), fibromyalgia (FM) and osteoarthritis (OA), experience fatigue [1, 2]. The causes of RMD-fatigue remain unclear, but the role of inflammation has been long, and fiercely, debated [3–, 4–9]. Fatigue reduces in response to anti-inflammatory treatments [9–11]. However it is common for fatigue to persist despite inflammatory disease remission [12, 13]. Furthermore, irrespective of treatment, people with RMDs often experience acute and rapid daily fluctuations in fatigue [3, 7, 10], which are described by patients as unpredictable, unearned and unfair [14–, 15–17].

Inflammation may cause fatigue directly (e.g. via sickness behaviour mechanisms, excess inflammatory cell populations and expression levels, or other disease-specific inflammatory pathways), or indirectly via its action on other common RMD co-morbidities such as pain and mood [4, 8, 9–, 18–22] which in turn increase fatigue. Understanding the relationship between inflammation, RMD symptoms, and fatigue requires a robust assessment of inflammatory pathways over multiple time-points to detect change in inflammation with contemporaneous remote-monitoring of fatigue (plus other potential explanatory factors) to detect within-person changes over time [18]. Logistically this has not been possible within traditional study designs.

A number of recent developments including our own successful use of smartphone apps to conduct ecological momentary assessment (EMA) studies which have collected serial assessments of symptoms at multiple time points per day and for an extended period (1–6 months) [23, 24], and remote dried blood spot sampling (DBSS) from which inflammatory markers could be extracted [25, 26] mean that these data could be collected. Crucially, these methods would allow researchers to more fully elucidate the relationship between fatigue, inflammation and a number of key covariates for the first time. Ultimately, the insights gained from applying these methods could mitigate the poor management of fatigue, and lead to the development of targeted treatment paradigms.

This study aimed to take advantage of these recent developments to determine the feasibility of embedding DBSS to examine inflammatory biomarkers among people with RMDs participating in a mHealth study.

## Methods

The “Gaining Insight into RheumAtic Fatigue” (GIRAF) study was advertised via online support groups, social media websites and public engagement portals, including People in Research (www.peopleinresearch.org), Twitter and Facebook. Further publicity was provided by charity partners Fibromyalgia Action UK (FMAUK; https://www.fmauk.org/) and the National Rheumatoid Arthritis Society (NRAS; https://www.nras.org.uk/) and the Manchester Research User Group (RUG) at the Centre for Musculoskeletal Research (http://www.cfe.manchester.ac.uk/connect/get-involved/rug/). Interested persons were asked to email the study team to obtain the study information sheet and link to the study’s screening questionnaire.

Eligible participants were aged 18 or older, with a primary diagnosis of RA, OA or FM and access to an Android 4.0 + or Apple (iOS 10 +) smartphone/tablet. Participants employed in a job that required night-shift work were excluded due to them having an alternative sleep–wake cycle. No further exclusion criteria were applied.

At least 24 h after returning the screening questionnaire potential participants were telephoned by KD to discuss the project. Verbal consent was obtained from those willing to participate and a study pack (including a form for written consent, a baseline questionnaire, DBSS kit and study instructions) was posted to participants. Participants received no additional training regarding the use of the app or DBSS kit, but were provided with contact information for the study team in case of problems or technical difficulties.

### Baseline questionnaire

A baseline questionnaire (Supplementary Material 1) was completed on, or before, the study start date. The questionnaire collected the following data:

#### Demographics

Participants reported their date of birth (DD/MM/YY), sex and employment status (see Supplementary Material 1). The age at which participants left education was recorded and categorised as those who completed secondary education (≤ 16 years) and those who completed further education (> 16 years). Participants’ postcodes were used to calculate levels of deprivation using either the English (2015 [27]) or Welsh (2019 [28]) Index of Multiple Deprivation. The month and year of disease onset was used to calculate disease duration.

### Daily monitoring

#### Symptom reports

Participants completed daily symptom monitoring by downloading and using uMotif, a patient co-designed smartphone/tablet app (www.umotif.com). uMotif has been used by a range of international academic and clinical organisations and we have previously shown high levels of app engagement among individuals with chronic pain and RA [23, 24]. Participants could install the app on any Android 4.0 + or Apple (iOS 10 +) smartphone or tablet, depending on their preferences.

For 30 days, participants received fixed interval-based prompts twice daily to complete 10 symptom ratings (supplementary material 2), on a 1–5 ordinal scale, once in the morning (8am) and once in the afternoon/evening (6 pm). Of those symptoms, the most relevant to determine rates of engagement and feasibility of study design were fatigue severity (1 = no fatigue, 5 = very severe fatigue), pain severity (no pain (1) to very severe pain (5)) and mood (depressed (1) to very happy (5)). In addition to the automatic data completion prompts, we undertook real-time data monitoring and targeted completion reminders, requesting data completion resume if the participant had not completed symptom reports for three or more days.

#### Dried Blood Spot Sampling (DBSS)

DBSS has been identified as an acceptable method of sample collection in epidemiological studies [25, 26, 29], which produces CRP values for which there is reasonable agreement with standard sampling by venepuncture [29]. Sampling is akin to how diabetics monitor blood sugar. Following the finger-prick, a blood droplet is allowed to form and dropped into a circle outlined on a protein saver card. Participants were asked to provide a minimum of 3 DBSS samples per card, to maximize the chance of receiving at least one viable sample per day. To provide the samples, participants were sent a kit comprising 10 each of safety lancets and protein saver cards (1 extra in case of sampling difficulties), 9 each of Silica desiccant sachets, foil pouches and business reply envelopes, and 1 disposable sharps bin (for disposal at a local pharmacy, or GP practice after study completion). Participants received reminders via the smartphone app to provide DBSS on days 1–7, 14 and 30. However, an issue in the system meant that reminders were not received between 09 – 12 and 22—29 March 2019. Nevertheless, reminders were sent on the majority of planned days (22/31 days, 71.0%), which translated into 234 (68.4%) of required samples being requested. Completion rates for all samples are compared to only those requested samples within the analysis. Due to the nature of DBSS sampling it was not possible to conduct real-time data monitoring and targeted completion reminders for DBSS.

Participants were provided with written instructions and a link to an instructional video (https://www.youtube.com/watch?v=he5D1LxbWdg), both of which had been designed in collaboration with people with RMDs at an earlier focus group, as part of patient and public involvement activities conducted to support this study.

Samples were returned, in pre-labelled, business reply envelopes, to study team members DG and KM, based at Ulster University, for analysis using established methods [29]. On the day of analysis, 3 mm paper discs were punched out of one of the received DBSS samples per day, and protein was extracted by addition of a routine elution buffer. DBSS extracts and plasma samples were logged, aliquoted and stored at -80’C until analysis. Samples were then analysed using an R&D Systems Quantikine ELISA to assess CRP concentrations according to manufacturer instructions. CRP values were converted from nanograms per milliliter (ng/ml) into milligrams per liter (mg/L), as used in clinics and compared to recognized “normal” values of CRP, considered to be CRP < 5 mg/L [30].

The reporting in this manuscript follows the CREMAS checklist for Ecological Momentary Assessment studies [31].

#### Analysis

We have previously shown that engagement with the uMotif app is high (89–91%) across a period of up to 6 months [23, 24]. Here, we determined the feasibility of embedding remote data collection of DBSS among people with RMDs participating in a mHealth study using the uMotif app. Specifically, we tested whether the inclusion of DBSS would decrease engagement.

##### Recruitment and attrition

We sought to recruit a total of 45 participants (15 each of RA, OA and FM) within a 2-week recruitment window. This sample size was determined pragmatically, given the exploratory nature of this study. Here, we report the number of people who a) completed the study’s screening questionnaire and provided consent for contact, b) were contacted to discuss participation c) were recruited and d) successfully installed the uMotif app and commenced data collection. The number of people who could not be included, and the reasons for exclusion are also reported.

##### Engagement with study app

Study engagement was first considered in terms of days on which symptoms (i.e. fatigue severity, pain and mood) were reported at least once (morning or evening). To inform future studies which aim to examine fluctuations in daily symptoms it is also important to understand engagement in terms of continuity of collected data. Continuity of symptom reports were examined graphically by plotting the symptom severity scores recorded for fatigue, pain and mood separately for each participant. In order to quantify continuity of symptom data we also calculated rates of engagement by determining the number of days on which within- and between-day changes in symptom severity could be calculated. Within-day changes in symptom severity values were calculated on days on which participants reported both morning and evening symptoms, at least once. Between day changes were calculated for both morning and evening assessments (e.g. Day 1 AM minus Day 2 AM; Day 1 PM minus Day 2 PM). We determined the proportion of days on which within- and between-day changes could be calculated, compared to the number expected, within higher values indicating greater continuity of symptom reporting.

No missing data imputation was undertaken and as a result, within-day changes could not be calculated for one participant who recorded only morning symptom assessments across their entire study period.

##### Feasibility of and engagement with DBSS

To determine whether DBSS was a feasible method of data collection we first determined the number of samples returned as a proportion of the samples expected across days 1–7, 14 and 30, irrespective of the number of DBSS reminders which were received by participants. To determine the proportion of eligible samples, from those expected, we then excluded any returned samples which appeared to be duplicates, or had missing/ incorrect sample dates (e.g. a sample provided on a date which did not match the expected dates for the participant).

As with the symptom reports, we determined continuity of samples to measure engagement. However, unlike the symptom reports, participants were not expected to complete DBSS on all days of the study. For that reason, examination of the continuity of DBSS completion is restricted to days 1–7. Continuity was first examined graphically, by plotting participants’ daily CRP (mg/L) scores. We then quantified continuity based on determining the number of between-day changes in CRP which could be calculated, compared to the number expected.

## Results

### Recruitment and attrition

A total of 73 persons completed the study’s screening questionnaire and provided consent for contact. Within the two-week recruitment window it was possible to contact the first 50 persons to discuss participation, and 44 people (13 RA, 15 OA, and 16 FM; 97.8% of target sample size) were recruited. Of those recruited, 42 (95.5%) successfully installed the uMotif app and commenced data collection. Four of those who installed the app did not return their study packs, did not therefore provide written consent, and were not eligible for the analysis.

In total 38 people (12 RA, 13 OA, and 13 FM; 84.4% of target sample size) were included in the study. The demographic characteristics for all participants, and by disease diagnosis, are shown in Table 1. Most of the participants were female (82%), with a  median age of 56 years. The majority (69%) of participants had completed further education (i.e. left education after 16 years old), and were either in full-time employment (24.3%), or retired (29.7%). There were no substantial differences (i.e. differences with distinct 95% CIs, or IQRs) between the disease groups (Table 1).

**Table 1:** Baseline characteristics

		All participants^a^ (*n* = 38)	RA (*n* = 12)	OA (*n* = 13)	FM (*n* = 13)
Female: *n*(%)		31 (82)	9 (75)	10 (77)	12 (92)
Age (years): median (IQR)		56 (43–65)	55(41–71)	64 (54–68)	47 (41–57)
Deprivation^b^: median (IQR)		7.0 (3.0–9.0)	8.0 (6.5–9.5)	6.0 (3.0–9.0)	6.0 (1.0–7.0)
Further education (> 16 years): n(%)		26 (68)	6 (50)	10 (77)	10 (77)
Employment status: *n*(%)	Full time	9 (24)	6 (50)	2 (15)	1 (8)
	Part-time	7 (19)	2 (17)	2 (15)	3 (25)
	Medically retired	8 (22)	1 (8)	3 (23)	4 (33)
	Retired	11 (30)	3 (25)	6 (46)	2 (17)
	Other	2 (5)	-	-	2 (7)
Duration of disease (years): median (IQR)		6.0 (3.0–12.0)	9.0 (5.5–10.5)	11.5 (5.0–28.0)	4.0 (2.0–6.0)

^a^includes all participants who were recruited, successfully installed the app and provided written consent to participate.

^b^Determined using the English (2015; *n* = 37) or Welsh (2014; *n* = 1) Index of Multiple Deprivation; 2occupation missing for 1 FM participant

### Study engagement

#### Symptom reporting: Fatigue, pain and mood

**Fig. 1:**
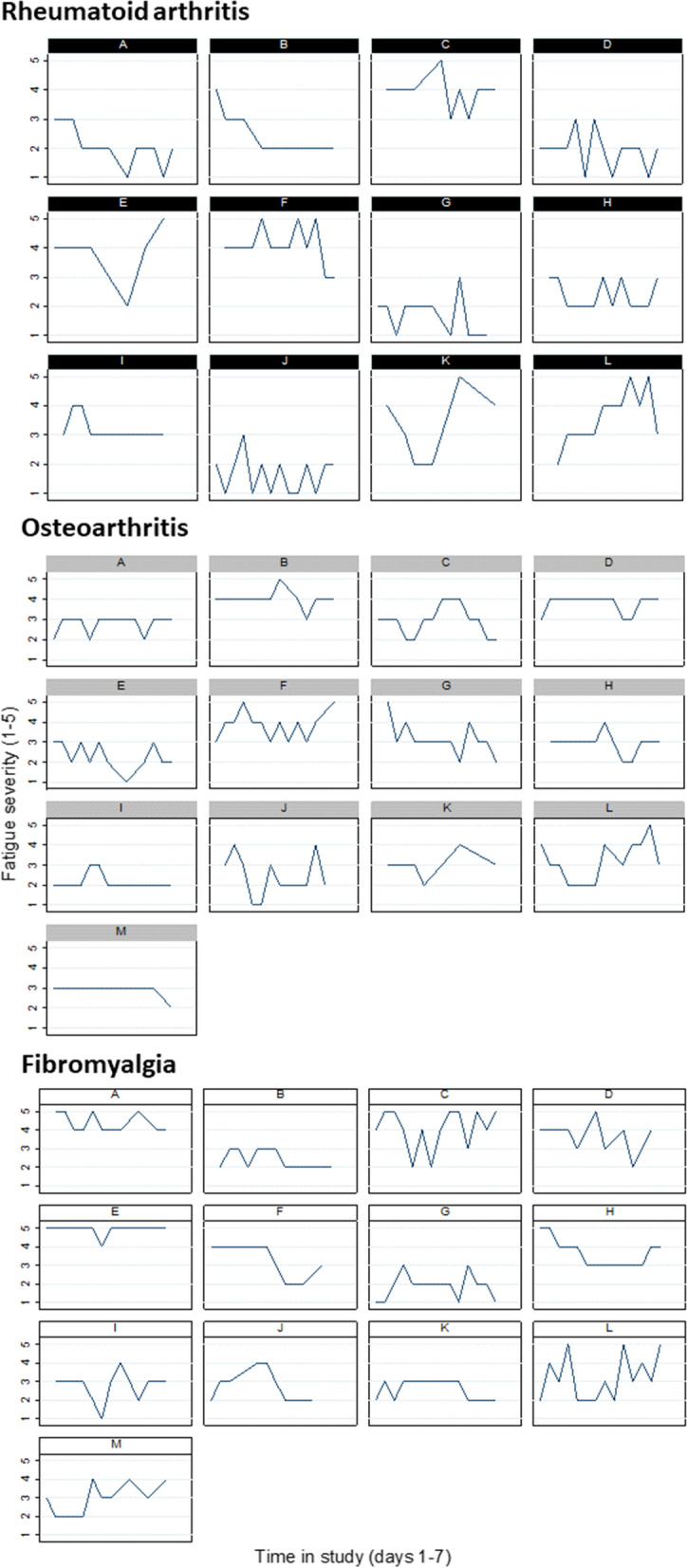
Fatigue severity scores (1 = no fatigue, 5 = very severe fatigue) reported on days 1–30Each graph represents an individual participant. Black: Rheumatoid Arthritis participants, Grey: Osteoarthritis participants, White: Fibromyalgia participants

Completion rates for reporting fatigue, pain and mood were high across the study. Continuity of symptom reporting was high (Fatigue: Fig. 1, Pain and Mood: see supplementary material 3).

Across 1140 study days (38 participants completing 30 days each), participants completed at least one of each symptom report on 95% of the days (fatigue: 1085 days, 95.2%; pain: 1083 days, 95.0%; mood: 1087 days, 95.4%).

Participants reported fatigue, pain or mood at least twice per day on approximately two-thirds of the study days (fatigue: 744 days, 65.3%; pain: 752 days, 66.0%; mood: 738 days, 64.7%). Finally, we calculated the proportion of between-morning and between-afternoon changes which could be calculated out of a maximum possible number of 1102 changes. Approximately two-thirds of between-morning changes in symptom severity could be calculated for all symptoms (fatigue: 744 days, 67.5%; pain: 743 days, 67.4%; mood: 731 days, 66.3%). The proportion of between-afternoon changes which could be calculated was slightly higher at approximately 70% for all symptoms (fatigue: 767 days, 70.0%; pain: 772 days, 70.1%; mood: 771 days, 70.0%).

### DBSS

Of 342 DBSS samples expected (38 participants completing 9 samples each), a total of 332 were received (97.1%). Of those, 45 samples were excluded from the analysis due to duplicate (*n* = 2) or missing/incorrect sample dates (*n* = 43). In total 287 (83.9% of those expected) samples were suitable for analysis and 100% of eligible samples were found to be viable for CRP extraction. Completion rates did not appear to be impacted by reminders not being sent (all sample completion: 83.9%, only samples requested by app: 83.8% (196/234)).

**Fig. 2:**
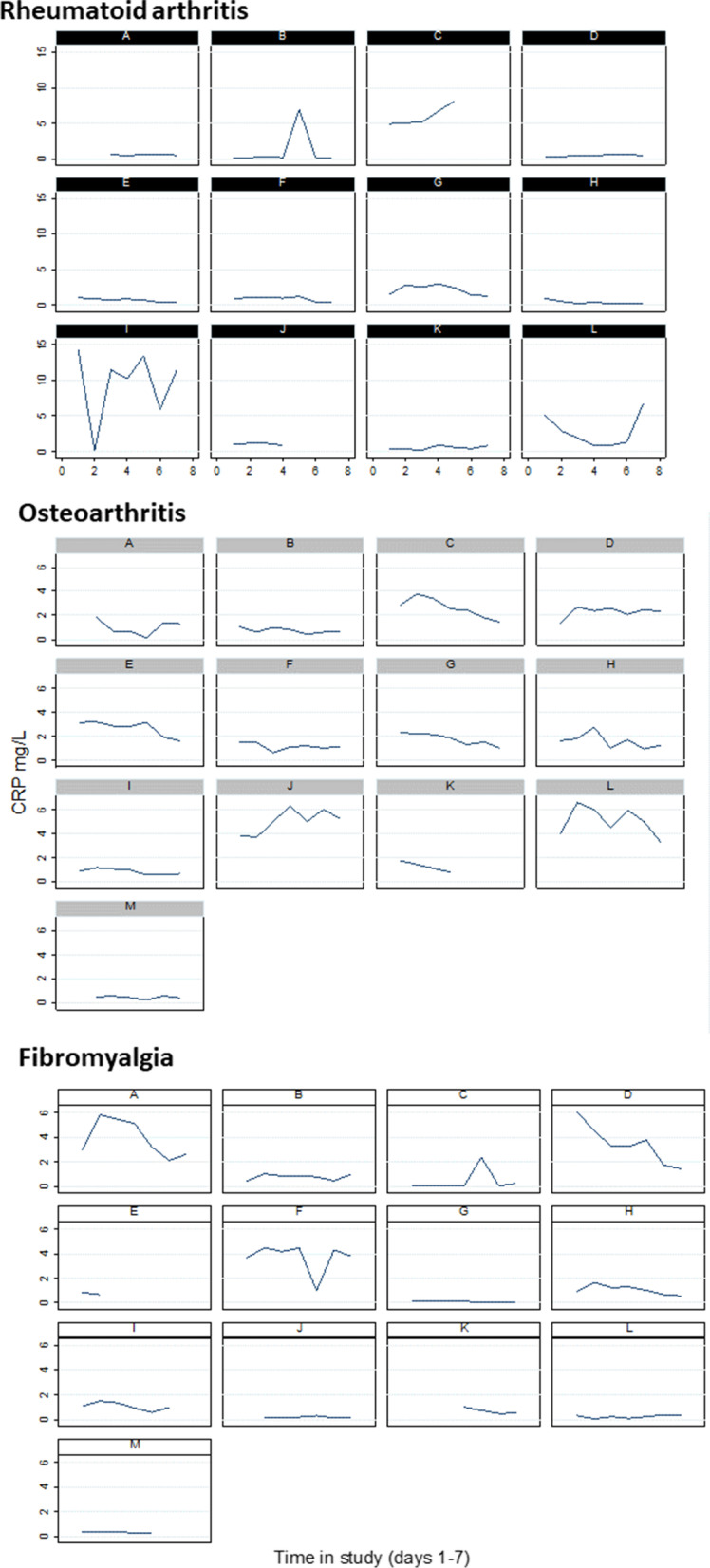
C-Reactive Protein values obtained from participants on days 1–7Each graph represents an individual participant. Black: Rheumatoid Arthritis participants, Grey: Osteoarthritis participants, White: Fibromyalgia participants

CRP levels were generally within normal range (< 5 mg/L [30]; 0.26–14.30 mg/L) throughout the first 7 days in the study (Fig. 2), with active inflammation (≥ 5 mg/L) observed in 22 of 234 samples (9.4%; 8 participants 4 RA, 2 OA, 2 FM). A total of 189 between day changes were calculated from a maximum 228 possible changes (82.9%). Daily changes in CRP ranged from 0.002–14.18 mg/L. Large changes in CRP were rare, daily changes > 5 mg/L occurring between 7 of 189 days (3.7%) in three participants with RA (25.0%; Fig. 2 RA panels B, I, L).

## Discussion

This feasibility study determined the viability of using DBSS as a method of remote blood sample collection among individuals with RMDs participating in a mHealth study. We have demonstrated that DBSS is a feasible tool for sample collection in RMD studies, observing high completion rates (≥ 83%) across 30 days, and full viability of samples returned to the study team. In parallel, engagement with the study app was high throughout the study (symptoms reported at least once on ≥ 95% of days). Thus, we are, to our knowledge, the first remote monitoring study to demonstrate successful engagement with the use of serial at-home blood sampling and daily symptom reporting among people with different rheumatic diseases.

We have also shown that individual patterns of fatigue, pain and mood varied substantially, but that sizeable changes in CRP were rare, with few people experiencing active inflammation (CRP > 5 mg/L) during the study period.

When interpreting these results, several limitations should be considered. First, the extent to which these results can be generalised to other populations is unclear. Due to the recruitment strategy adopted our population are self-selected. This may mean that our high completion rates are a result of recruiting those who are more likely to be engaged with the study. However, the rates of engagement observed are comparable with our previous study which used the uMotif app in a Chronic Pain population for up to one year [23, 24], indicating that data collection using this platform is highly successful. Similarly, due to the recruitment strategy used, we were unable to validate patient reported diagnoses and this may have led to misclassification, or ineligible participants joining the study. However, this study did not seek to determine the precise nature of the role between inflammation and fatigue in clinically confirmed RMD patients. Rather, it was designed to determine whether individuals reporting an RMD condition could reasonably be expected to use DBSS in a future (fully remote) mobile health study. As a result, there is no reason to assume that these results will not be applicable to the populations who we anticipate recruiting into our main study.

Second, we selected CRP as our measure of inflammation here because a) it is a measure typically used in clinical assessments and research studies and b) analysis of CRP is cost-effective in a feasibility study such as ours. While we have shown that it is possible to extract CRP values using DBSS, we also showed that there was little variance in CRP despite high variance in fatigue. We note that this, in conjunction with our self-selection recruitment process, may suggest that (particularly for RA participants) we have recruited only those who are healthy and who have well controlled disease. However, it may also suggest that alternative fatigue-specific inflammatory markers (e.g. TNF-α, IL-1, IL-6 and IFN-γ [18, 32–36]) may better account for variation in fatigue, and we have not ascertained how viable DBSS is for their extraction. However, there is no plausible reason why this method of sample collection could not be used to extract other potential markers in a larger cohort in the future.

Similarly, while we collected a comprehensive set of questions within our feasibility study (not all data shown), fatigue is likely the product of a complex interaction between multifactorial contributors, including disease processes, feelings and behaviours and personal factors [4, 37]. As the causes of fatigue may differ between individuals and over time, it will be crucial that the mechanisms of fatigue be elucidated within a well-designed and well-powered longitudinal study which can capture fluctuations in both fatigue and its many possible causes.

Finally, this study was designed to test the feasibility of DBSS and so, although we had high rates of data completion, the sample size was small. This precluded formal examination of any relationships between fatigue, pain, mood and CRP and limits the conclusions which can be drawn from this dataset. Nevertheless, this study provides evidence to support the use of DBSS within a larger population that would be better positioned to determine the relationship between inflammation and fatigue, to test whether any association was causal, and to identify factors that may mediate those causal pathways. Conducting such an investigation would contribute to the development of intervention strategies to improve the clinical management of fatigue in patients with rheumatic diseases.

## Conclusion

Recent developments in remote data collection have provided exciting opportunities to obtain frequent and repeated measures for a range of self-report data. Here, we have shown that DBSS is a viable method of objective sample collection for use in mHealth studies. This enables researchers to obtain the serial assessments of symptoms and biological samples necessary to address hitherto unanswerable questions, such as elucidating the mechanisms of fatigue fluctuations.

## Supplementary Information

**Additional file 1. **Baseline Questionnaire.

**Additional file 2.** Symptoms recorded via the uMotif app.

**Additional file 3.** Symptom reporting: Pain and mood.

## Data Availability

The datasets used and/or analysed during the current study are available from the corresponding author on reasonable request.

## Abbreviations

CRP: C-Reactive Protein
DBSS: Dried Blood Spot Sampling
FM: Fibromyalgia
FMAUK: Fibromyalgia Action UK
GIRAF: Gaining Insight into RheumAtic Fatigue
OA: Osteoarthritis
NRAS: National Rheumatoid Arthritis Society
RA: Rheumatoid Arthritis
RMD: Rheumatic and Musculoskeletal Diseases
RUG: Research User Group
