# Supplementary material for: Remote sampling of biomarkers of inflammation with linked patient generated health data in patients with rheumatic and musculoskeletal diseases: an Ecological Momentary Assessment feasibility study
Source: BMC Musculoskelet Disord. 2022 Aug 13;23:770. doi: 10.1186/s12891-022-05723-w (PMC9375303; doi:10.1186/s12891-022-05723-w)
Supplement: Supplementary file 1 — Additional file 1. Baseline Questionnaire. [file 12891_2022_5723_MOESM1_ESM.pdf]

GIRAFid: | | | |

GIRAF

Health Questionnaire

Thank you for agreeing to take part in the GIRAF study. This questionnaire will ask you about a number of issues in three sections:

1. Demographic information
2. Information about your rheumatic disease
3. Health status information

Your answers are very important to us. All information you give us will be kept strictly confidential and individual participants will not be identifiable in any published results.

Thank you for your help,

The GIRAF study team

Please enter the date you completed this questionnaire: D D M M Y Y

If you have any questions, contact:

Katie Druce Tel: 0161 275 1604

Email: katie.druce@manchester.ac.uk

MANCHESTER 1824

The University of Manchester

Section 1

Please try to answer every question in the booklet. There are no right or wrong answers. Please note all instructions are in dark coloured boxes. We would like to ask you some questions about your demographic information.

Please answer the questions by crossing the box ☒ that you think most closely applies to you.

1 **Are you?**

Male ☐ Female ☐

2 **What is your date of birth?**
e.g. 12th August 1945 should be written as 12 08 1945

☐☐ Day ☐☐ Month ☐☐☐☐ Year

3 **What is the first part of your postcode?**

☐☐☐☐

4 **What is your height?**
You may answer in metric or imperial.

☐☐☐ cm or ☐ ft ☐☐ ins

5 **What is your weight?**
You may answer in metric or imperial.

☐☐☐ kg or ☐☐ st ☐☐ lbs

6 **How would you describe your ethnic origin?**

☐

7 **At what age did you leave full time education?**

☐

8 **What is your smoking status?**.

☐ Current smoker ☐ Ex-smoker ☐ Never smoker

9 **What is your occupational status?**

- Working full time (≥30 hours per week) ☐
- Working part time (<30 hours per week) ☐
- Student (full-time or part-time) ☐
- Medically retired ☐
- Voluntary worker ☐
- Unemployed but seeking work ☐
- Retired ☐

10 **What is your marital status?**

- Single ☐
- In a relationship ☐
- Co-habiting ☐
- Married ☐
- Civil partnership ☐
- Separated ☐
- Divorced ☐
- Widowed ☐

11 **On average, how many units of alcohol do you drink per week?**

- 0 ☐
- 1-5 ☐
- 6-10 ☐
- 11-15 ☐
- 16-20 ☐
- 21-40 ☐
- >40 ☐

As a guide:

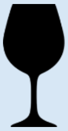
1 medium wine (175ml)
2.1 units

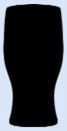
1 pint of beer/lager/cider
3 units

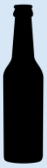
1 bottle of beer/lager/cider
1.7 units

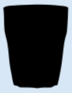
1 single 25ml shot of spirit
1 unit

Section 2

We would like to ask you some questions about your rheumatic disease (RD).

14 **Which of the following is your primary diagnosis**.

- Rheumatoid arthritis ☐
- Osteoarthritis ☐
- Fibromyalgia ☐

15 **When was the approximate date when you received your primary diagnosis (MM/YY)?**
e.g. August 2007 should be written as 08 07

☐☐ Month ☐☐ Year

Please answer the questions by crossing the box ☒ that you think most closely applies to you.

16 **In addition to your primary diagnosis, have you ever been diagnosed with any of The following?** Please tick as many as apply to you.

Rheumatoid arthritis	☐	Diabetes	☐
Osteoarthritis	☐	Thyroid disorder	☐
Fibromyalgia	☐	Multiple sclerosis	☐
Spondyloarthropathy/ ankylosing spondylitis	☐	Hypertension	☐
Gout, or other Crystal arthritis	☐	Depression	☐
Sjogren's Syndrome	☐	None of the above	☐

Other Diagnoses, please describe:

17 Which, if any, of the following are you currently taking?

Painkillers

Paracetamol ☐

NSAIDs
e.g. ibuprofen, diclofenac, naproxen, indomethacin, meloxicam, ketoprofen, celecoxib, etoricoxib, etodolac ☐

Other analgesics
e.g. *cocodamol, codydramol* ☐

Weak opiates
e.g. codeine, dihydrocodeine, tramadol ☐

Strong opiates
e.g. buprenorphine, fentanyl, morphine, oxycodone ☐

Drugs for neuropathic pain
e.g. gabapentin, pregabalin, duloxetine ☐

Disease modifying anti-rheumatic drugs (DMARDs)

Glucocorticoids (or steroids)
e.g. prednisolone, methylprednisolone, dexamethasone ☐

Synthetic DMARDs
e.g. Methotrexate, sulfasalazine, hydroxychloroquine, leflunomide, gold, azathioprine, mycofenolate, cyclosporine, cyclophosphamide ☐

Biologic DMARDs
e.g. Etanercept (Enbrel), infliximab (Remicade), adalimumab (Humira), certolizumab (Cimzia), golimumab (Simponi), rituximab (Mabthera), tocilizumab (Actemra), abatacept (Orencia) ☐

Sleep medications

Sedatives (or hypnotics)
e.g. diazepam (Valium), Temazepam (Normison/Euhypnos), Zopiclone (Zimovane) Nitrazepam (Mogadon/Nitrados) ☐

Mood stabilisers
e.g. Lithium (Priadel/Camcolit), socium valproate (Epolim), Carbamazepine (Tergretol, Teril, Epimaz ☐

Antidepressants
e.g. Paroxetine (Seroxat), Fluoxetine (Prozac), Citalopram (Cipramil), duloxetine (Cymbalta/Yentreve) Amitriptyline (Elavil/Tryptizol/Lentizol) ☐

Other sleep mediactions
e.g. Chlorpromazine (CPZ/Largactil), Haloperidol (Haldol/Serenace), Quetiapine (Seroquel), Risperidone (Risperdal ☐

18 **Are you currently using other medications or coping strategies?**

Yes ☐ 1 No ☐ 0

Please use the below space to record any additional medications or non-pharmacological coping strategies you use.

Medication/coping strategies	Why do you take the medication or use the coping strategies?

Section 3

We would like to know how fatigue has affected you in the past 7 days. Please answer all of the questions. Don't think too long and hard, just give your first reaction – there are no right or wrong answers!

19 **Please circle the number that shows your average level of fatigue during the past 7 days.**

0	1	2	3	4	5	6	7	8	9	10

No fatigue — Totally exhausted

For each of the following questions, please tick one answer that best applies to you.

20 **How many days did you experience fatigue during the past week (7 days)?**

- 0 ☐
- 1 ☐
- 2 ☐
- 3 ☐
- 4 ☐
- 5 ☐
- 6 ☐
- Every day ☐

20 **How long, on average, has each episode of fatigue lasted during the last 7 days?**

- Less than an hour ☐
- Several hours ☐
- All day ☐

Over the past 7 days……

		Not at all	A little	Quite a bit	Very much
21	Have you lacked physical energy because of fatigue?	☐	☐	☐	☐
22	Has fatigue made it difficult to bath or shower?	☐	☐	☐	☐
23	Has fatigue made it difficult to dress yourself?	☐	☐	☐	☐
24	Has fatigue made it difficult to do your work or other daily activities?	☐	☐	☐	☐

Over the past 7 days......

		Not at all	A little	Quite a bit	Very much
25	**Have you avoided making plans because of fatigue?** *e.g. plans to go out, or do jobs around the home or garden*	☐	☐	☐	☐
26	**Has fatigue affected your social life?**	☐	☐	☐	☐
27	**Have you cancelled plans because of fatigue?** *e.g. plans to go out, or do jobs around the home or garden*	☐	☐	☐	☐
28	**Have you refused invitations because of fatigue?** *e.g. meeting up with a friend*	☐	☐	☐	☐
29	**Have you lacked mental energy because of fatigue?**	☐	☐	☐	☐
30	**Have you forgotten things because of fatigue?**	☐	☐	☐	☐
31	**Has fatigue made it difficult to think clearly?**	☐	☐	☐	☐
32	**Has fatigue made it difficult to concentrate?**	☐	☐	☐	☐
28	**Have you made mistakes because of fatigue?**	☐	☐	☐	☐
29	**Have you felt you have less control in areas of your life because of fatigue?**	☐	☐	☐	☐
30	**Have you felt embarrassed because of fatigue?**	☐	☐	☐	☐
31	**Has being fatigued upset you?**	☐	☐	☐	☐
32	**Have you felt down or depressed because of fatigue?**	☐	☐	☐	☐

We would like to know more about any problems you have had with feeling tired, weak or lacking in energy in the last month. Please answer ALL the questions by ticking the answer which applies to you most closely. If you have been feeling tired for a long while, then compare yourself to how you felt when you were last well. Please tick only one box per line.

		Less than usual	No more than usual	More than usual	Much more than usual
33	Do you have problems with tiredness?	☐	☐	☐	☐
34	Do you need to rest more?	☐	☐	☐	☐
35	Do you feel sleepy or drowsy?	☐	☐	☐	☐
36	Do you have problems starting things?	☐	☐	☐	☐
37	Do you lack energy?	☐	☐	☐	☐
38	Do you have less strength in your muscles?	☐	☐	☐	☐
39	Do you feel weak?	☐	☐	☐	☐
40	Do you have difficulties concentrating?	☐	☐	☐	☐
41	Do you make slips of the tongue when speaking?	☐	☐	☐	☐
42	Do you find it more difficult to find the correct word?	☐	☐	☐	☐

		Better than usual	No worse than usual	Worse than usual	Much worse than usual
43	How is your memory?	☐	☐	☐	☐

44 **Have you had any problems with your energy levels for 6 months or longer?**

Yes ☐ 1 No ☐ 0

We would like to ask you some questions about your functional ability and general health
Please cross the box ☒ that is closest to your abilities **during the past four weeks**

45 **How often were you physically able to drive a car or use public transportation?**

- All days ☐
- Most days ☐
- Some days ☐
- Few days ☐
- No days ☐

46 **How often were you in a bed or chair for most of the day?**

- All days ☐
- Most days ☐
- Some days ☐
- Few days ☐
- No days ☐

47 **Did you have trouble doing vigorous activities such as running, lifting heavy objects, or participating in strenuous sports?**

- All days ☐
- Most days ☐
- Some days ☐
- Few days ☐
- No days ☐

48 **Did you have trouble either walking several blocks or climbing a few flights of stairs?**

- All days ☐
- Most days ☐
- Some days ☐
- Few days ☐
- No days ☐

During the past four weeks

49 **Were you unable to walk unless assisted by another person or by a cane, crutches or walker?**

- All days ☐
- Most days ☐
- Some days ☐
- Few days ☐
- No days ☐

50 **Could you easily write with a pen or pencil?**

- All days ☐
- Most days ☐
- Some days ☐
- Few days ☐
- No days ☐

51 **Could you easily button a shirt or blouse?**

- All days ☐
- Most days ☐
- Some days ☐
- Few days ☐
- No days ☐

52 **Could you easily turn a key in a lock?**

- All days ☐
- Most days ☐
- Some days ☐
- Few days ☐
- No days ☐

53 **Could you easily comb or brush your hair?**

- All days ☐
- Most days ☐
- Some days ☐
- Few days ☐
- No days ☐

54 **Could you easily reach shelves that were above your head?**

- All days ☐
- Most days ☐
- Some days ☐
- Few days ☐
- No days ☐

55 **Did you need help to get dressed?**

- All days ☐
- Most days ☐
- Some days ☐
- Few days ☐
- No days ☐

56 **Did you need help to get out of bed?**

- All days ☐
- Most days ☐
- Some days ☐
- Few days ☐
- No days ☐

During the past four weeks

57 How often did you have severe pain from your arthritis?

- All days ☐
- Most days ☐
- Some days ☐
- Few days ☐
- No days ☐

58 How often did your morning stiffness last more than one hour from the time you woke up?

- All days ☐
- Most days ☐
- Some days ☐
- Few days ☐
- No days ☐

59 How often did your pain make it difficult for you to sleep?

- All days ☐
- Most days ☐
- Some days ☐
- Few days ☐
- No days ☐

60 How often have you felt tense or high strung?

- All days ☐
- Most days ☐
- Some days ☐
- Few days ☐
- No days ☐

61 How often have you been bothered by nervousness or your nerves?

- All days ☐
- Most days ☐
- Some days ☐
- Few days ☐
- No days ☐

62 How often have you been in low or very low spirits?

- All days ☐
- Most days ☐
- Some days ☐
- Few days ☐
- No days ☐

63 How often have you enjoyed the things you do?

- All days ☐
- Most days ☐
- Some days ☐
- Few days ☐
- No days ☐

64 How often did you feel like a burden to others?

- All days ☐
- Most days ☐
- Some days ☐
- Few days ☐
- No days ☐

During the past four weeks

65 **How often did you get together with friends or relatives?**

- All days ☐
- Most days ☐
- Some days ☐
- Few days ☐
- No days ☐

66 **How often were you on the telephone with close friends or relatives?**

- All days ☐
- Most days ☐
- Some days ☐
- Few days ☐
- No days ☐

67 **How often have you enjoyed the things you do?**

- All days ☐
- Most days ☐
- Some days ☐
- Few days ☐
- No days ☐

68 **How often did you feel like a burden to others?**

- All days ☐ 0
- Most days ☐ 1
- Some days ☐ 2
- Few days ☐ 3
- No days ☐ 4

69 How often did you get together with friends or relatives?

- All days ☐
- Most days ☐
- Some days ☐
- Few days ☐
- No days ☐

70 How often were you on the telephone with close friends or relatives?

- All days ☐
- Most days ☐
- Some days ☐
- Few days ☐
- No days ☐

71 How often did you go to a meeting of a place of worship, club, team, or other groups?

- All days ☐
- Most days ☐
- Some days ☐
- Few days ☐
- No days ☐

72 Did you feel that your family or friends were sensitive to your personal needs?

- All days ☐
- Most days ☐
- Some days ☐
- Few days ☐
- No days ☐

During the past four weeks

73 How often were you unable to do any paid work, house work or school work?

- All days ☐
- Most days ☐
- Some days ☐
- Few days ☐
- No days ☐

74 On the days you did work, how often did you have to work a shorter day?

- All days ☐
- Most days ☐
- Some days ☐
- Few days ☐
- No days ☐

Please cross the box ☒ that is closest to how you have been feeling in the past week

75 I feel tense or wound up

- Most of the time ☐
- A lot of the time ☐
- From time to time ☐
- Not at all ☐

76 I feel as if I am slowed down

- Most of the time ☐
- A lot of the time ☐
- From time to time ☐
- Not at all ☐

77 I still enjoy the things I used to

- Definitely as much ☐
- Not quite so much ☐
- Only a little ☐
- Hardly at all ☐

78 I get a sort of frightened feeling like ‘butterflies’ in the stomach

- Most of the time ☐
- A lot of the time ☐
- From time to time ☐
- Not at all ☐

79 I get a sort of frightened feeling as if something awful is about to happen

- Very definitely and quite badly ☐
- Yes, but not too badly ☐
- A little, but it doesn’t worry me ☐
- Not at all ☐

80 I have lost interest in my appearance

- Definitely ☐
- I don’t take as much care as I should ☐
- I may not take quite as much care ☐
- I take just as much care as ever ☐

81 I can laugh and see the funny side of things

- As much as I always could ☐
- Not quite so much now ☐
- Definitely not quite so much now ☐
- Not at all ☐

In the past week

82 **I feel restless as I have to be on the move**

- Very much indeed ☐
- Quite a lot ☐
- Not very much ☐
- Not at all ☐

83 **Worrying thoughts go through my mind**

- A great deal of the time ☐
- A lot of the time ☐
- From time to time, but not too often ☐
- Only occasionally ☐

84 **I look forward with enjoyment to things**

- As much as I ever did ☐
- Rather less than I used to ☐
- Definitely less than I used to ☐
- Hardly at all ☐

85 **I feel cheerful**

- Not at all ☐
- Not often ☐
- Sometimes ☐
- Most of the time ☐

86 **I get sudden feelings of panic**

- Very often indeed ☐
- Quite often ☐
- Not very often ☐
- Not at all ☐

87 I can sit at ease and feel relaxed

- Definitely ☐
- Usually ☐
- Not often ☐
- Not at all ☐

88 I can enjoy a good book or radio or TV program

- Often ☐
- Sometimes ☐
- Not often ☐
- Very seldom ☐

We would like to ask you some questions about your pain.

89 Throughout our lives, most of us have had pain from time to time (such as minor headaches, sprains, and toothaches). Have you had pain other than these everyday kinds of pain today?

No ☐ Yes ☐

90 On the diagram, shade in the areas where you feel pain. Put an X on the area that hurts the most.

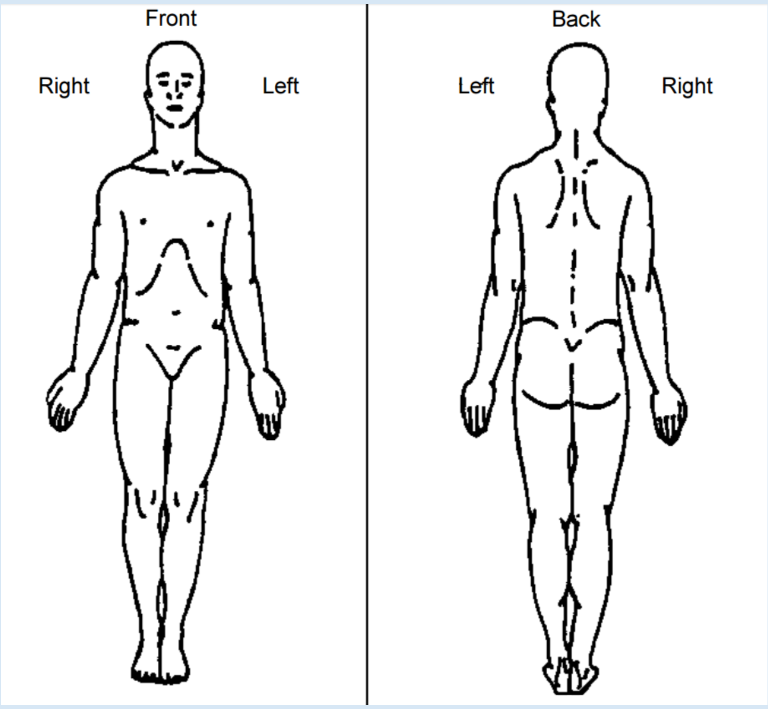

91 **Please rate your pain by marking the box beside the number that best describes your pain at its WORST in the past 24 hours.**

☐ 0 ☐ 1 ☐ 2 ☐ 3 ☐ 4 ☐ 5 ☐ 6 ☐ 7 ☐ 8 ☐ 9 ☐ 10 ☐

No pain — Pain as bad as you can imagine

92 **Please rate your pain by marking the box beside the number that best describes your pain at its LEAST in the past 24 hours.**

☐ 0 ☐ 1 ☐ 2 ☐ 3 ☐ 4 ☐ 5 ☐ 6 ☐ 7 ☐ 8 ☐ 9 ☐ 10 ☐

No pain — Pain as bad as you can imagine

93 **Please rate your pain by marking the box beside the number that best describes your pain on the AVERAGE.**

☐ 0 ☐ 1 ☐ 2 ☐ 3 ☐ 4 ☐ 5 ☐ 6 ☐ 7 ☐ 8 ☐ 9 ☐ 10 ☐

No pain — Pain as bad as you can imagine

94 **What treatments or medications are you receiving for your pain?**

95 **In the past 24 hours, how much RELIEF have pain treatments or medications provided? Please mark the one percentage that most shows how much relief you have received.**

☐ 0% ☐ 10% ☐ 20% ☐ 30% ☐ 40% ☐ 50% ☐ 60% ☐ 70% ☐ 80% ☐ 90% ☐ 100%

No Relief — Complete Relief

Mark the box beside the number that describes how, during the past 24 hours, pain has interfered with your:

96 **General Activity**

☐ 0 ☐ 1 ☐ 2 ☐ 3 ☐ 4 ☐ 5 ☐ 6 ☐ 7 ☐ 8 ☐ 9 ☐ 10 ☐

Does not interfere — Completely interferes

Mark the box beside the number that describes how, during the past 24 hours, pain has interfered with your:

97 **Mood**

0 1 2 3 4 5 6 7 8 9 10

Does not interfere — Completely interferes

98 **Walking ability**

0 1 2 3 4 5 6 7 8 9 10

Does not interfere — Completely interferes

99 **Normal work (includes both work outside the home and housework)**

0 1 2 3 4 5 6 7 8 9 10

Does not interfere — Completely interferes

100 **Relations with other people**

0 1 2 3 4 5 6 7 8 9 10

Does not interfere — Completely interferes

101 **Sleep**

0 1 2 3 4 5 6 7 8 9 10

Does not interfere — Completely interferes

102 **Enjoyment of life**

0 1 2 3 4 5 6 7 8 9 10

Does not interfere — Completely interferes

Copyright statements

Questions 63-76
HADS copyright © R.P. Snaith and A.S. Zigmond, 1983, 1992, 1994. Record form items originally published in Acta Psychiatrica Scandinavica, 67, 361–70, copyright © Munksgaard International Publishers Ltd, Copenhagen, 1983. This edition first published in 1994 by nferNelson Publishing Company Ltd, 389 Chiswick High Road, London W4 4AJ GL Assessment is part of GL Education www.gl-assessment.co.uk This form may not be reproduced by any means without first obtaining permission from the publisher.
Email: permissions@gl-assessment.co.uk

GL Assessment

Thank you for completing this questionnaire.

Please now place the questionnaire to in the envelope provided and send along with one copy of the consent form and the activity monitor after 30 days.

GIRAF
