# Supplementary material for: Remote sampling of biomarkers of inflammation with linked patient generated health data in patients with rheumatic and musculoskeletal diseases: an Ecological Momentary Assessment feasibility study
Source: BMC Musculoskelet Disord. 2022 Aug 13;23:770. doi: 10.1186/s12891-022-05723-w (PMC9375303; doi:10.1186/s12891-022-05723-w)

Symptoms recorded via the uMotif app

| **Name** | **Question** | **1** | **2** | **3** | **4** | **5** |
| --- | --- | --- | --- | --- | --- | --- |
| **Fatigue Severity** | How severe is your fatigue? | No Fatigue | Mild fatigue | Moderate fatigue | Severe fatigue | Very Severe Fatigue |
| **Fatigue Impact** | How much impact is fatigue having on your life? | No impact | Mild impact | Moderate impact | Severe impact | Very Severe impact |
| **Fatigue Coping** | How well are you coping with your fatigue? | Not at all well | Not well | Moderately well | Quite well | Very well |
| **Pain** | How severe is your pain? | No Pain | Mild pain | Moderate pain | Severe pain | Very Severe Pain |
| **Mood** | How is your mood? | Depressed | Feeling low | Not very happy | Quite happy | Very Happy |
| **Wellbeing** | How well do you feel? | Very Well | Quite well | Moderately well | Quite unwell | Very Unwell |
| **Anxiety** | How anxious do you feel? | Not Anxious | Mildly anxious | Moderately anxious | Anxious | Very Anxious |
| **Illness Impact** | How much is your illness impacting on your activities? | No Impact | Mild impact | Moderate impact | Severe impact | Very Severe Impact |
| **Disease Control** | How much control do you feel you have over your symptoms? | No Control | Little control | Moderate control | Good control | Very Good Control |
| **Concentration** | How would you rate your concentration? | Poor | Bad | Moderate | Good | Excellent |

uMotif platform as seen by participants


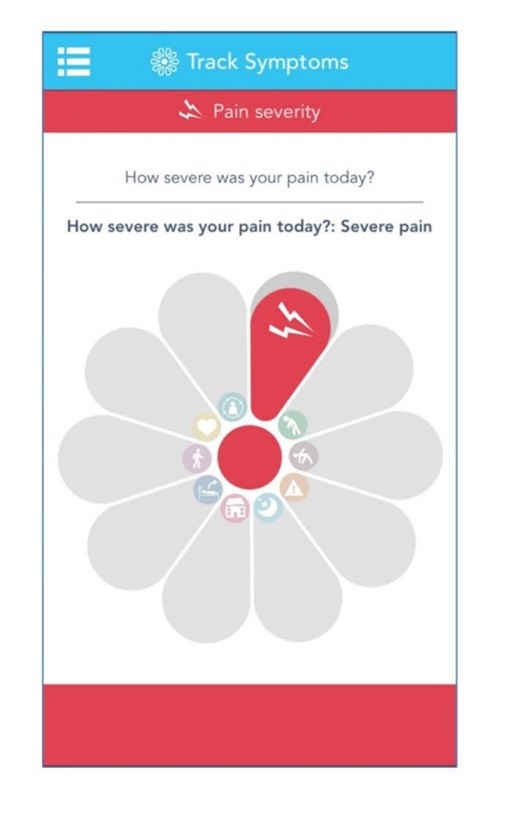

Supplement: Supplementary file 2 — Additional file 2. Symptoms recorded via the uMotif app. [file 12891_2022_5723_MOESM2_ESM.docx]
