# Supplementary material for: Remote sampling of biomarkers of inflammation with linked patient generated health data in patients with rheumatic and musculoskeletal diseases: an Ecological Momentary Assessment feasibility study
Source: BMC Musculoskelet Disord. 2022 Aug 13;23:770. doi: 10.1186/s12891-022-05723-w (PMC9375303; doi:10.1186/s12891-022-05723-w)
Supplement: Supplementary file 3 — Additional file 3. Symptom reporting: Pain and mood. [file 12891_2022_5723_MOESM3_ESM.docx]

Symptom reporting: pain


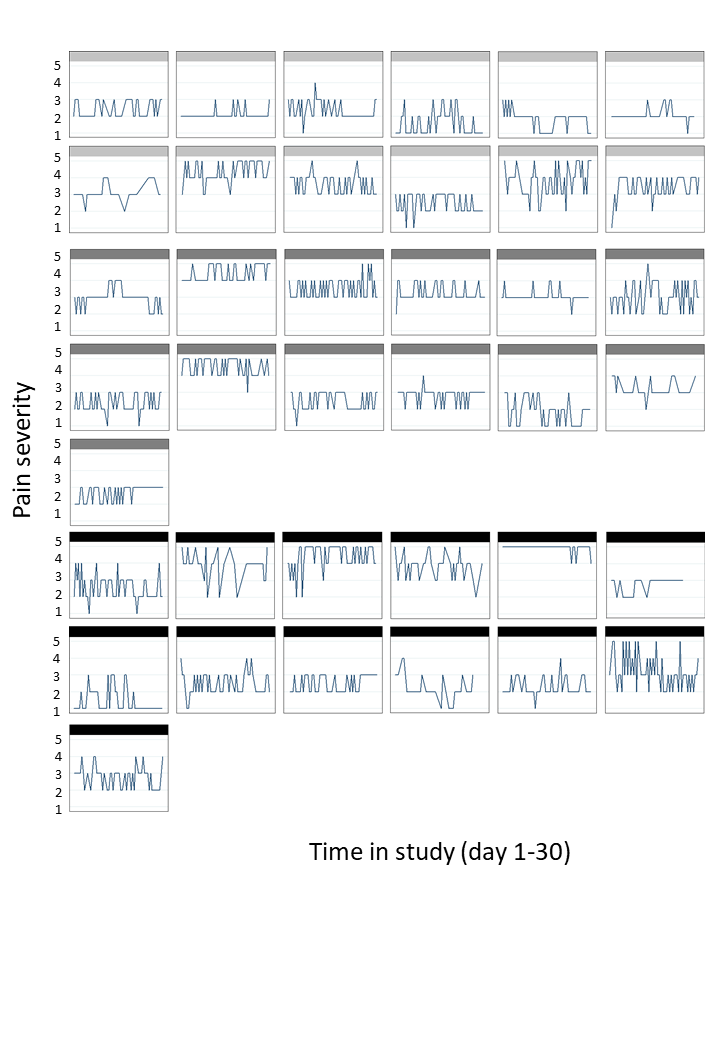


Figure 1– Patterns of pain severity during the study. Each graph represents an individual participant. 1= no pain, 5= very severe pain.

Symptom reporting: mood


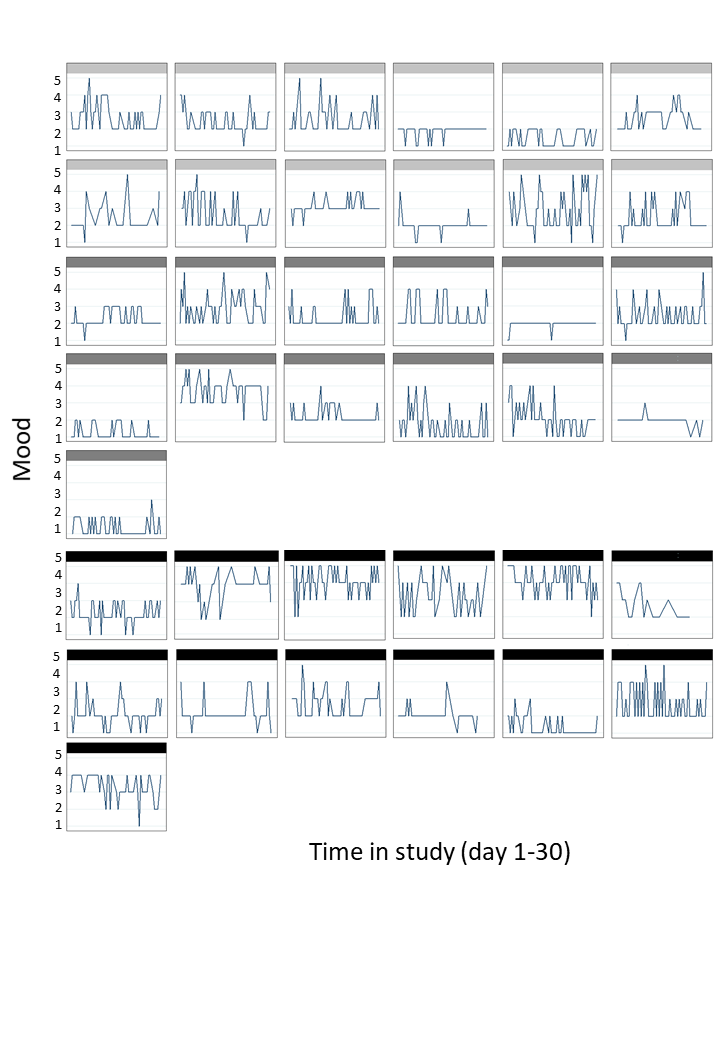
Figure 2 – Patterns of mood during the study. Each graph represents an individual participant. 1= depressed, 5= very happy.
